# Role of the Sterol Regulatory Element Binding Protein Pathway in Tumorigenesis

**DOI:** 10.3389/fonc.2020.01788

**Published:** 2020-09-08

**Authors:** Tao Jiang, Guangji Zhang, Zhaohuan Lou

**Affiliations:** ^1^School of Basic Medical Sciences, Zhejiang Chinese Medical University, Hangzhou, China; ^2^College of Pharmaceutical Sciences, Zhejiang Chinese Medical University, Hangzhou, China

**Keywords:** natural drug, sterol regulatory protein, lipid metabolism, glucose metabolism, microenvironment, cytoprotection

## Abstract

Metabolic changes are a major feature of tumors, including various metabolic forms, such as energy, lipid, and amino acid metabolism. Sterol regulatory element binding proteins (SREBPs) are important modules in regulating lipid metabolism and play an essential role in metabolic diseases. In the previous decades, the regulatory range of SREBPs has been markedly expanded. It was found that SREBPs also played a critical role in tumor development. SREBPs are involved in energy supply, lipid supply, immune environment and inflammatory environment shaping in tumor cells, and as a protective umbrella to support the malignant proliferation of tumor cells. Natural medicine and traditional Chinese medicine, as an important part of drug therapy, demonstrates the multifaceted effects of SREBPs regulation. This review summarizes the core processes in the involvement of SREBPs in tumors and provides a comprehensive understanding of the pathways through which natural drugs target the SREBP pathway and regulate tumor progression.

## Introduction

Tumors are a major health challenge for humanity, with high annual mortality rates. In the continuous efforts to discover new treatments for tumors, metabolic changes have been receiving considerable attention. Metabolic changes involve numerous forms, including energy, sugar, lipids, and amino acids. Differing from that of normal cells, tumor metabolism is unique. As early as 1920, a study conducted by Warburg found that tumor cells obtained energy in a glycolysis anoxic environment (1). This led many scholars to focus on the direction of energy metabolism change and use it as a key point for tumor treatment (2). The massive proliferation of tumors requires a variety of substances, including energy, lipids, etc. To cope with these additional needs, tumor cells undergo extensive metabolic reprogramming (3–6). As a component of cellular basal metabolism, lipid metabolism plays a very important role in the metabolic programming of tumor cells (7). Lipids, including phospholipids and cholesterol, serve as a major component of the cell membrane and an additional energy supply to the cells (8–10). In addition, they play important biological regulatory roles as signaling molecules, specific receptors, and transcription factors (11–13). Growing evidence shows that tumor cells are involved in a large number of changes in lipid metabolism, which has become one of the important features of tumors (3, 7, 14). This also renders the key molecules of lipid metabolism and targeting the regulation of lipid metabolism a promising strategy for the treatment of tumors (15, 16).

For most normal cells, the growth rate is strictly regulated by the body, and lipids derived from *de novo* synthesis and dietary intake of hepatocytes can meet their growth needs. However, for proliferating malignant tumor cells, this is not sufficient; hence, the process of extra lipid production is activated in tumor cells (17). The production of fat involves multiple successive biological steps that can be controlled by numerous regulatory factors and different key enzymes. These enzymes and factors have exhibited a strong association with tumors. For example, high levels of fatty acid synthase (FASN) expression have been associated with invasive tumor phenotypes (18, 19), while both acetyl-CoA carboxylase and FASN have been shown to be highly expressed in malignant tumors and are also indicators of poor prognosis (7, 20, 21). These enzymes are regulated by a variety of complex mechanisms, and a large number of studies have shown that SREBPs are important molecules regulating these key enzymes and leading to lipid metabolism disorders (22–27). Many natural drugs can regulate SREBPs and various biological processes involved in different pathways. This review summarizes the key processes involved in targeting SREBPs through natural drugs for the treatment of tumors.

## Overview of SREBPs

**FIGURE 1:**
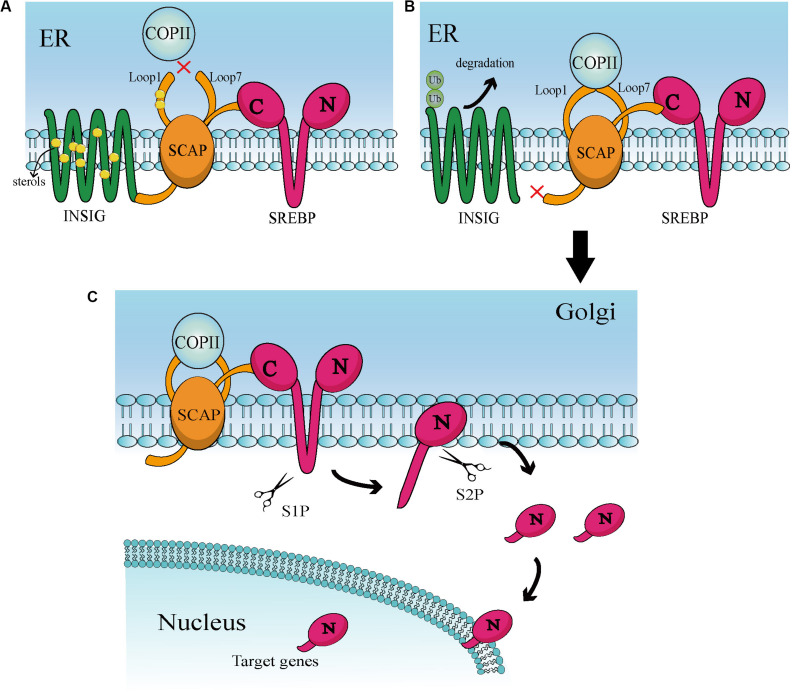
**(A)** At high sterol levels, the sterol binds to Loop 1 and induces conformational changes in SCAP, thereby increasing its affinity for INSIG. Then, binding of SCAP and INSIG triggers a second conformational changes in SACP, leading to the dissociation of Loop1 and Loop7. This conformation prevents COPII from binding to SCAP. In addition, sterol is combined with INSIG to keep the stability of INSIG. These interactions help the INSIG/SCAP/SREBP complex to be preserved on the ER membrane. **(B)** In a sterol-deprived environment, the reduction of sterol leads to ubiquitination and rapid degradation of INSIG. This gives the SCAP/SREBP complex an opportunity to escape ER. Deprivation of sterols also triggers conformational changes that enable Loop1 to bind to Loop7, which allows SCAP to bind to COPII. Then, COPII transports the complex to the Golgi apparatus via vesicles. **(C)** When in the Golgi, SREBP will be continuously cleaved by S1P and S2P proteases, ultimately releasing the N-terminal active domain. This active part enters the nucleus and binds to numerous target genes of SREBP, leading to downstream effects.

Sterol regulatory element-binding proteins (SREBPs) belong to a small family of membrane-bound proteins, and are basic helix-loop-helix leucine zipper transcription factors. There are three subtypes, namely SREBP-1a, SREBP-1c, and SREBP-2. Of those, SREBP-1a and SREBP-1c are encoded by the same gene, whereas SREBP-2 is encoded by a different gene (28). SREBP-1 is mainly regulated by caloric restriction (29, 30), while SREBP-2 is stimulated by thyroid hormone and itself (31, 32). SREBP-2 also preferentially participates in gene transcription in cholesterol biosynthesis (26, 33). Under physiological conditions, activation of SREBPs is tightly regulated by a negative feedback loop triggered by sterols in the endoplasmic reticulum (ER) (28, 34). The classical activation is mediated mainly by insulin-induced gene (INSIG) and SREBP cleavage-activating protein (SCAP) (Figure 1). Specifically, the SREBPs precursor protein transforms to a complex with another ER localized protein, termed SCAP. This complex interacts with the INSIG1 and INSIG2 proteins (35, 36). When the levels of cellular cholesterol are high, INSIGs become stable and allow the SCAP-SREBP complex to preserve in the ER (28, 37). When the levels of sterols in the ER decrease below the threshold, INSIGs are ubiquitylated and rapidly degraded (38) that can trigger the isolate of SCAP-SREBP complex from the ER (39). The isolated complex cannot be transported directly to the Golgi and needs specialized transport vesicles generated by coatomer complex II (COPII). In this process, the levels of sterol will lead to conformation changes in SCAP to determine whether SCAP can combine with COPII (40). These factors together lead to the transport of the SCAP-SREBP complex from the ER to Golgi. In the Golgi, SREBPs will be consecutively cleaved by two membrane-bound proteases site-1 protease (S1P) (41) and site-2 protease (S2P) (42). Then, cleaved SREBPs release the transcriptionally active NH2-terminal domains, that can enter into the nucleus and induce target gene expression including the SREBPs transcription factor itself (25, 43), consequently causing a series of downstream changes.

In addition to the strict restriction of transport and activation, SREBP is transcriptionally regulated through several mechanisms. As mentioned above, mature NH2-terminal domains of SREBPs enhances expression of itself (31). Liver X receptor (LXR) is one of the key molecules mediating SREBP mRNA transcription (44). Studies have shown that LXR plays an important role in tumors and is associated with SREBPs (45, 46). The same effect was observed for insulin via the mTOR pathway mechanism (47). Moreover, several miRNAs including miRNA-29, miRNA-185, and miRNA-342, are also responsible for the transcription of SREBP (48, 49).

Sterol regulatory element-binding proteins are involved in numerous biological processes, such as ER stress, inflammation, autophagy, and apoptosis. This also causes SREBPs to trigger a variety of diseases, including obesity, dyslipidemia, diabetes, non-alcoholic fatty liver disease, non-alcoholic steatohepatitis, chronic kidney disease, neurodegenerative diseases, and tumors (50). In the previous decades, the definitive function of SREBPs has been extended to many tumor-critical biological processes, highlighting the importance of lipids in cell and system homeostasis (19, 22). In an epidemiological study, long-term intake of cholesterol was also associated with an increased risk of gastrointestinal cancer (51). This suggests that uptake of exogenous lipids also exerts an effect on tumor progression. Multiple pathways mediated by SREBPs are associated with lipid metabolism and play a role in tumor progression. Although the sterol feedback regulation mechanism of SREBPs has been extensively studied, the mechanism through which SREBPs induce lipid metabolism and other specific regulatory functions in tumors remains unclear (52).

## Drug Family Targeting SREBPs

In view of the special and powerful regulation of SREBPs in tumors, drugs targeting SREBPs have been developed. However, direct inhibition of SREBPs is a difficult task, as transcription factors are difficult to target for drugs (2). The current strategy is to inhibit the transport of SREBPs from the ER to Golgi and inhibit cleavage enzymes to block the release of the active domain. Besides this, inhibiting SREBPs transcription through key signaling pathways and restoring cholesterol sensitivity by inhibiting cholesterol droplets formation are also common interventions (53). Table 1 shows some of the common inhibitors of SCAP/SREBPs. Most of them are still in the preclinical stage, and a few of them have been studied clinically.

**TABLE 1:** The classical inhibitors targeting SCAP/SREBPs in cancer cells.

Compounds/drugs	Mechanism	Preclinical	References	Clinical trial	References
Fatostatin	Inhibition of SCAP/SREBP transportation	GBM Prostate Lung Pancreatic Endometrial	(54) (55–57) (58) (59) (60, 61)	None	N/A
PF-429242	Inhibition of SREBP cleavage by inhibiting SIP	Liver GBM Pancreatic	(62) (63) (59)	None	N/A
Nelfinavir	Inhibition of SREBP cleavage by inhibiting S2P	Liposarcoma Prostate	(64) (65)	Rectal Myeloma Lung Pancreatic	(66) (67) (68) (69)
1,10-phenanthroline	Inhibition of SREBP cleavage by inhibiting S2P	Prostate	(65)	None	
Docosahexaenoic acid	Inhibition of the transcription of SREBPs	Breast	(70)	Melanoma Breast	(71) (72)
Ursodeoxycholic acid	Inhibition of the transcription of SREBPs	Liver	(73)	Colorectal Esophageal ALL	(74) (75) (76)
BF175	Inhibition of the transcription of SREBPs	None	N/A	None	N/A
25-hydroxycholesterol	Inhibition of SCAP/SREBP transportation	GBM	(77)	None	N/A

*GBM, glioblastoma; ALL, acute lymphoblastic leukemia.*

Although there have been many classical inhibitors, their application is still limited. SREBP inhibitors, including fatostatin, BF175, and 25-hydroxycholesterol cholesterol are only observed in a pre-clinical study. In part, these classical SREBP inhibitors such as 25-hydroxycholesterol, also activate LXR while inhibiting SREBPs, which will reactivate the SREBP-1c gene as a feedback loop. This can lead to enhanced expression of free fatty acid synthesis genes and increased plasma triacylglycerol content, thereby greatly limiting drug performance (78). Some drugs have entered clinical trials, but the analysis of SREBP is not sufficient. For example, nelfinavir is widely used in cancer research, but its main research course is to inhibit the Akt signaling pathway (79). Therefore, we need to find some emerging SREBPs inhibitors to avoid these side effects. Natural drugs have garnered increasing attention given their lower cost and minor side effects. Many of them are used in metabolic diseases and have shown the ability to inhibit SREBPs. Table 2 shows some of the natural drugs involved in the regulation of SREBPs, as well as a brief description of their mechanism of action. These compounds can target SACP/SREBPs, inhibit the detachment of SREBPs from the ER, inhibit the transport of SREBPs, and regulate the key molecular mechanisms involved in SREBPs. Although there are only few studies on tumors, they can serve as a guide for drug research and can be candidates for tumor treatment.

**TABLE 2:** Natural drugs targeting SREBPs and their mechanism of action.

Compounds/drugs	Effects	Mechanism	References	Clinical trials
Xanthohumol	Inhibition of SREBP cleavage	Competitive combination with S1P	(80, 81)	Prevents DNA Damage by Dietary Carcinogens
Betulin	Inhibition of SREBPs transcription	Promoting the combination of SCAP and INSIGs	(82)	Wound healing (83) Actinic keratoses (84)
Silibinin	Inhibition of SREBPs nuclear translocation	Increasing SREBP1 phosphorylation by AMPK	(85)	NAFLD (86) Hepatitis (87) Breast cancer (88) Preeclampsia (89)
3,5-dicaffeoyl-epi-quinic acid	Inhibition of SREBPs transcription	Activating AMPK/MAPK signaling pathway	(90)	None
Salvianolic acid	Inhibition of SREBPs transcription	Blocking STAT-3/SREBP1 signaling	(91)	Hepatitis (92)
Long leaf mantle extract	Inhibition of SREBPs transcription	Activating Wnt/β-catenin pathway	(93)	None
Paeoniflorin	Inhibition of SREBPs transcription	Activating LKB1/AMPK signaling pathway	(94)	Rheumatoid Arthritis (95)
RA-XII	Inhibition of SREBPs transcription	Inhibiting SCAP protein	(96, 97)	None

*mTOR, mammalian target of rapamycin; S1P, sphingosine 1-phosphate; AMPK, activated protein kinase; MAPK, mitogen activated protein kinase; STAT-3, signal transducer and activator of transcription 3; Keap1, Kelch like ECH associated protein 1; Nrf2, nuclear respiratory factor 2; SCAP, SREBP cleavage-activating protein; PSC, primary sclerosing cholangitis.*

The mechanism of these drugs is multi-targeted and involves a variety of common signaling pathways. Their impact is also multifaceted, involving a variety of biological processes, such as cellular lipid supply, energy supply, glucose supply, cell protection, and microenvironment modeling. In the following sections, we provide a more detailed description of the biological functions involved in the regulation of SREBPs by natural medicines.

## Regulation of Tumor Cell Energy Supply

Cells require energy to maintain their vitality, growth, and normal physiological functions (98). ATP is the currency of cell energy supply (99). In normal cells, ATP is produced by glycolysis in the cytoplasm, oxidative phosphorylation in mitochondria, tricarboxylic acid recycling, β-oxidation of fatty acid, and the metabolism of ketones and triglycerides also produces energy. The energy supply of normal cells is mostly provided by the aerobic oxidation of glucose, while in the tumor-associated microenvironment it is mainly provided through aerobic glycolysis (1). Aerobic glycolysis results in a small amount of ATP and a large number of intermediate products required for cell proliferation (6, 100, 101). Recent research showed that SREBP-1 plays an important role in the regulation of lipid metabolism and contributes substantially to glucose metabolism (102); there exists an interactive relationship between SREPBs and glucose. SREPBs are necessary for intracellular glycolysis and oxidative phosphorylation in natural killer cells (103). SREBP-1a can *trans-*regulate the promoter of the PFKFB gene (104), which is a 6-phosphate fructose-2-kinase/fructose-2,6-bisphosphate enzyme catalyzing the synthesis of fructose-2,6-diphosphate and degradation in gluconeogenesis. SREBP-1 is also involved in the generation of glycogen under special situation. The lack of SREBP-1 reduces the levels of glycogen and lower the activity of glycogen synthase mRNA (105). On the contrary, glucose exerts a reverse regulatory effect on SREBPs. Kinetic experiments showed that exogenous glucose can upregulate SREBP-1c precursors and ribosomes within 30 min following the translocation of SREBP-1c to the nucleus. Glucose rapidly stimulates SREBP-1c maturation through the Janus kinase/signal transducer and activator of transcription pathway (106). Meanwhile, the protein SCAP binding SREBP in the ER is also stimulated by glucose, leading to glycosylation of SCAP and further promoting the release of mature SREBPs (107). Collectively, these studies have shown that SREBPs, especially SREBP-1 and SREBP-1c, play important roles in regulating glucose metabolism. Aerobic glycolysis is widespread in tumor cells. Current studies indicate that SREBPs may affect the progression of tumors by altering glucose metabolism (108). Some natural drugs, such as silibinin, can affect glucose uptake through SREBP-phosphatidylinositol 3 kinase-protein kinase B (109), and paeoniflorin can promote β-oxidation and glycogen production (94) to regulate energy metabolism. These compounds may be the potential candidates for the treatment of tumors through regulation of energy metabolism.

## Regulating FAO as Supplementary Energy

Aerobic glycolysis is an inefficient oxidation method associated with limited energy supply. Therefore, tumor cells have to take various measures, such as increasing the rate of glycolysis to produce lactic acid (110) and strengthening fatty acid oxidation (FAO) (111) to produce more ATP. FAO is an important auxiliary production mode of the body. In the case of nutritional deficiency, FA replaces glucose to provide sufficient energy for the body. Meanwhile, FAO is also the preferred way of energy supply for the heart, skeletal muscle, and kidneys (112). Studies have found changes in FAO in various types of tumors. In triple-negative breast tumors, FAO is extremely active, and blocking FAO may greatly influence energy metabolism, reduce proliferation, and inhibit growth *in vivo* in tumor cells (113). Similar results were also reported in models of prostate cancer, multiple myeloma, and leukemia (114–117). Moreover, FAO was also the main energy provider in metastatic tumors (111). As the primary energy supplier for some cancer cells, the process of FAO is affected by SREBPs. The basis of FAO is long-chain FA, which can be obtained through food or synthesized endogenously. SREBPs (especially SREBP-1) upregulate a large number of enzymes that catalyze the synthesis of FA, such as FASN, and stearoyl coenzyme α desaturating enzyme in a variety of human tumors (118–120). Silencing of SREBP-1 or SREBP-2 in established tumor cell lines and tumor cells of patient origin led to an overall transformation of cell metabolism, including glycolysis, mitochondrial respiration, and reduction of the levels of FAO (108). In the natural drug family ursodeoxycholic acid can regulate the expression of SREBPs and the occurrence of FAO, thereby improving inflammatory response, angiogenesis, and macrophage differentiation (121). These biological processes exert marked effects in tumors and affect tumor progression. Therefore, natural drugs may be worthy of study in regulating SREBPs and pointing to the process of FAO in blocking the development of tumors.

## Providing the Necessary Lipids for the Proliferation of Tumor Cells

Lipids are particularly important for maintaining the biosynthesis of cell membranes and coordinating numerous biological processes (13, 50, 122). Phospholipids are widely involved in the construction of cell membrane modules (123), while cholesterol is one of the main components of lipid rafts that can be used as the tissue center for the assembly of signaling molecules (12). This is necessary for cell division, metabolism, and proliferation (124, 125). Unlike normal cells that obtain lipids from the blood in the form of dietary free FAs, tumor cells show a strong *de novo* synthesis of lipids to support their growth (126).

**FIGURE 2:**
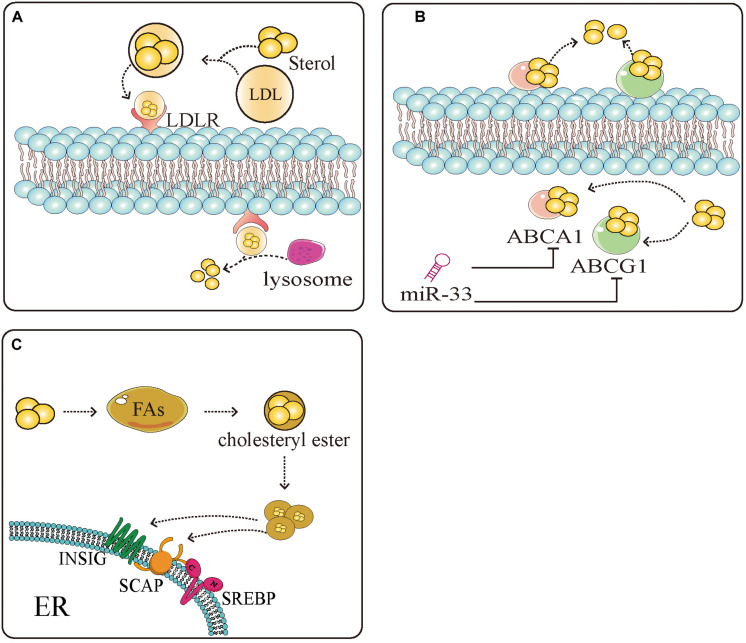
Sterol regulatory element-binding protein (SREBPs) mediate the uptake, efflux, and storage of cholesterol. **(A)** Extracellular cholesterol is carried by low density lipoprotein (LDL) and binds to LDL receptors (LDLR) on the cell surface. After binding, cholesterol is transported to the cells and decomposed by the lysosome into intracellular cholesterol. **(B)** Intracellular cholesterol binds to the sterol transporter ATP binding cassette subfamily A member 1 (ABCA1)/ATP binding cassette subfamily G member 1 (ABCG1) and is transferred to the extracellular space to complete the uptake and discharge of cholesterol. miR-33 which is embedded in introns of, and co-transcribed with SREBPs can inhibit the expression of ABCA1 and ABCG1, thus inhibiting the reversal of cholesterol. **(C)** Excess cholesterol in the cells binds to SREBP-mediated fatty acids and esterifies them into cholesterol esters to avoid the negative regulation of cholesterol. These measures provide a large amount of energy and nutrients and protection for tumor cell proliferation.

A large amount of lipid production in tumor cells is mediated by SREBPs. SREBPs regulate the production of sterols, especially cholesterol. Studies have shown that tumor cells possess high cholesterol levels, derived by increasing the uptake of low-density lipoprotein (LDL), reducing the outflow of cholesterol, and accelerating endogenous synthesis of cholesterol and FAs (Figure 2) (127–130). SREBPs are involved in almost all the pathways associated with high cholesterol. They increase the levels of cholesterol in cells by increasing the intake of LDL and synthesis of cholesterol. Inhibition of low-density lipoprotein receptor (LDLR) can promote glioblastoma cell death (46). In addition, they promote the transcription of enzymes, such as 3-hydroxy-3-methyl-glutaryl-CoA reductase (HMGCR), that can directly induce the synthesis of cholesterol (131). Meanwhile, miR-33 which is embedded in introns of, and co-transcribed with SREBPs can also prevent loss of cholesterol by inhibiting the expression of cassette subfamily transporters (e.g., ABCA1 and ABCG1) to antagonize the binding between transporters and cholesterol (132). Overaccumulation of unesterified cholesterol can be toxic to cells. Cholesterol esters, which mainly exist as cytosolic lipid droplets, can be a safe form to store cholesterol. Increased lipid droplets have been found in glioblastoma and some other types of tumor (34, 133). Tumor cells maintain a large amount of cholesterol by combining cholesterol and FA to the formation of cholesterol ester to escape the monitoring of cholesterol (134). Abnormal levels of lipids are intimately related to carcinogenesis and cancer metastasis (135). In liver cancer, obesity caused by the accumulation of lipids accelerates the progression of hepatitis and liver cancer. It was also found that an increase in total cholesterol can lead to the development of gastric cancer (136). Natural drugs have great potential in maintaining lipid homeostasis. Schisandra polysaccharide can improve the production of lipids by downregulating SREBP-2/HMGCR (137). Xanthohumol is a natural inhibitor of SREBPs that competes with sphingosine-1-phosphate to antagonize the activation of SREBPs inhibiting the synthesis of cholesterol (81). Similarly, betulin, ursodeoxycholic acid, and other natural drugs possess unique properties, inhibiting SREBPs to regulate lipid homeostasis (82, 138). The production of lipids, especially cholesterol, can be limited by natural drugs through the SREBPs pathway, suggesting that the effects of natural drugs in altering the production of lipids may be applied to the treatment of tumors.

Currently, there is insufficient evidence regarding the regulation of phospholipid production by SREBPs. However, recent studies have provided clues concerning the interaction between SREBPs and phospholipids. In small intestinal tumors, phospholipid remodeling can change the expression of SREBPs to regulate the generation of cholesterol, and affect the occurrence and development of intestinal tumors (139). Low levels of phosphatidylcholine lead to the maturation of SREBP-1 in nematode or mammalian models (140), and phospholipids rich in eicosapentaenoic acid inhibit the SREBP-1c-mediated production of fat. These findings suggest a relationship between phospholipids, cholesterol, and SREBPs.

## Provide Protection for Tumor Cells

Sterol regulatory element-binding protein-mediated regulation of lipids provides considerable protection for tumor cells. Improving drug resistance and regulating cell cycle are common ways. Research has found that statins exert a good effect on some prostate tumors (141, 142); however, this effect can be blocked by SREBPs. Studies have shown that SREBPs may lower the sensitivity of statins in the treatment of prostate tumors by upregulating HMGCR and other lipid metabolism genes (143). Hence, inhibition of HMGCR and SREBPs would increase responsiveness to drugs (144). The same drug resistance mechanism related to SREBPs and lipid metabolism was also found in breast tumors, multiple myeloma, glioblastoma, lung tumors, and liver tumors (22, 58, 77, 145–147), as well as anti-tumor drugs, including cisplatin (148), rapamycin, epidermal growth factor receptor-targeted inhibitors and docetaxel (57, 147, 149). Natural drugs combined with traditional anti-tumor drugs to increase the sensitivity of cells to treatment are very promising. In the treatment of hepatocellular carcinoma, emodin has been used to increase the sensitivity of cells to sorafenib by regulating SREBP-2 (150). Although the evidence is promising, further research studies are warranted to investigate the mechanism of SREBPs involved in drug resistance.

In addition to participating in the development of drug resistance, SREBP-mediated metabolism of glycolipids also protects tumor cells in multiple aspects. SREBP-2-mediated synthesis of sterol protects against oxidative stress by reducing lipid peroxidation to maintain membrane integrity (151). SREBP-2 also occupies the promoter of autophagy-related genes to activate autophagy (152). Cells can remove damaged proteins and organelles through autophagy, while recapturing energy and essential substances through the same process during periods of nutrient deficiency (153). SREBPs can also promote tumorigenesis by activating the mevalonate pathway (154). SREBPs lead to the *de novo* synthesis of lipids; however, excess lipids do not cause negative feedback regulation with SREBPs, because lipids are stored in cells in the form of lipid droplets. Lipid droplets have been observed in many tumors and may become a potential biomarkers in tumor (133). Reduction of LD formation by inhibiting SOAT1 can effectively suppress tumor growth (53). These lipids can help in the late stage of cell metabolism and counteract the lipid toxicity induced by the accumulation of intracellular FA (155, 156).

Sterol regulatory element-binding proteins can also directly or indirectly regulate the cell cycle to facilitate the proliferation of tumor cells. Indirectly, the SREBP-mediated synthesis of FAs and cholesterol has a certain impact on the cell cycle. Unsaturated FAs increase the expression of cyclin D1 and cell proliferation by activating β-catenin in renal clear cell carcinoma (157). Cholesterol is also essential for cell cycle progression, and cholesterol deficiency leads to cell cycle arrest at the G2/M phase (124). Directly, SREBP-1 contains a binding site in the host cell factor C1 gene, through which it stimulates the expression of key genes involved in cell cycle control and participates in the cell cycle and fibroin A adjustment (158). Studies have also shown that silencing SREBP-1 can directly lead to cell arrest at the G1 phase in human HeLa, U2OS, and MCF-7 cells, thereby attenuating cell growth (159). However, different subtypes of SREBPs appear to play different roles in the regulation of the cell cycle. Unlike SREBP-1, excess SREBP-1a causes cell cycle disorder, resulting in the accumulation of cyclin-dependent kinase inhibitors, such as p27, p21, and p16. Moreover, overexpression of SREBP-1a activates a novel SREBP binding site in the promoter of the gene p21 (waf1/cip1) that activates cyclin-dependent kinase inhibitors, leading to inhibition of cell growth and cell cycle arrest at the G1 phase (160, 161). SREBP-1c also promotes cell cycle progression by enhancing the expression of its target gene pituitary tumor-transforming 1 (162). Pituitary tumor-transforming 1 prevents premature chromosome segregation by inhibiting the activity of isolated enzymes, and promotes cell cycle disorders by regulating cell cycle genes (163). Previous research shed light on the effects of SREBPs on the regulation of tumor cell cycle. However, further studies area warranted to completely elucidate the mechanism involved in this process.

## Potential in Modulating Inflammation and Immunity

**FIGURE 3:**
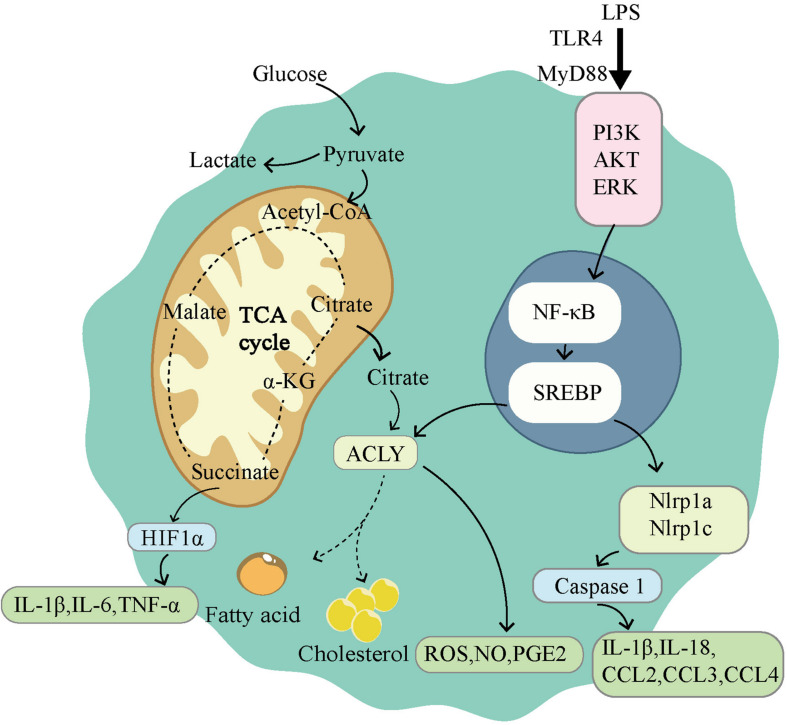
Sterol regulatory element-binding protein (SREBPs) is involved in the activation of M1-type macrophages. M1 macrophages use glycolysis as the main way of energy supply. The tricarboxylic acid cycle is disrupted in M1 macrophages, leading to the accumulation of citrate and succinate. Succinate can activate HIF1α, which in turn leads to the release of proinflammatory factors. LPS induces the activation of NF-κB through TLR4 dependent and independent pathways. Activation of NF-κB induces the expression of SREBPs, promoting lipid synthesis and accumulation. ACLY, as a downstream gene of SREBP, participates in lipid synthesis and drives the release of ROS, NO, PGE2. SREBPs also activate Nlrp1a and Nlrp1c, which lead to the release of proinflammatory factors.

The immune environment and inflammatory environment are hotspots of the tumor microenvironment. This environment can be shaped in many ways; and metabolism plays a unique role (164). As a transcription factor that modulates a large number of metabolism-related genes, SREBPs show great potential in shaping the tumor microenvironment. Although current research has focused on cardiovascular and adipose diseases, we predict that the same results may be achieved in tumors. Here, we review the known studies of SREBP with the aim to provide assistance for future treatment. We first focused on macrophages because they are important cells for intervention in inflammation and immunity. As one of the important immune cells of the body, macrophages show powerful phagocytosis and SREBPs exert control by influencing the expressions of target genes. As early as the 1970s, changes in the composition of membrane FAs have been shown to affect phagocytosis by macrophages (165). The phagocytic immunity of macrophages depends on the direct interaction between the plasma membrane and the actin cytoskeleton (166). SREBP-1a can regulate a variety of lipid components involved in the actin cytoskeleton network and cytoplasmic membrane to change the phagocytic function of macrophages (47). Moreover, macrophages exhibit significant plasticity and can be transformed into M1 and M2 phenotypes. The polarization change between M1 and M2 is an important intervention point for many diseases, including tumors. The two main phenotypes of macrophages showed distinct metabolic characteristics, and SREBPs are mainly responsible for the activation of M1 macrophages (Figure 3). M1 macrophages are known to rely on aerobic glycolysis. This metabolic adaptation favors rapid ATP production to sustain their phagocytic function and provides metabolic precursors to feed the pentose phosphate pathway. The levels of ATP citrate lyase (ACLY) and FAS are important in M1 macrophage activation. The increase of ACLY was found in activated M1 macrophages, and the silencing of ACLY was sufficient to reduce the expression of inflammatory mediators (167). Meanwhile, fatty acid synthase (FAS) deletion in macrophages prevented macrophage recruitment and inflammatory response in diabetic mice (168). Both ACLY and FAS are important partners of SREBP. Therefore, SREBP1-a was found to be highly expressed in the LPS-induced M1 macrophage model, and the deficiency of SREBP 1-a would lead to deficiency of innate immune response (169). On the contrary, M2 macrophages have an enhanced fatty acid oxidation (FAO) and oxidative phosphorylation (170). Blocking FAO by drugs inhibits IL-4-induced M2 polarization (171). The regulation of SREBPs involves a variety of lipids, which are the raw materials of FAO. In conclusion, SREBP can influence macrophage polarization and phagocytosis by conditioning its target genes. The consequence is inflammation and immune changes.

The effect of SREBPs was not restricted to macrophages alone. SREBPs is also widely involved in T-cell function and specific functions of innate and adaptive immunity (172). SREBPs are required for the metabolic reprogramming of mitotic signaling in response to CD8^+^ T cells. Loss of SREBPs in CD8^+^ T cells renders them ineffective for blast, resulting in decreased proliferative capacity *in vitro* and attenuated clonal expansion during viral infection (173). In dendritic cells, the accumulation of cholesterol accelerates the development of autoimmunity at the transcriptional level via the nod-like receptor 3 (NLRP3) isoform (174, 175). NLRP3 is an important molecule involved in the inflammatory response and innate immune response of the body. The immune response affected by NLRP3 *in vivo* or *in vitro* requires the participation of the SCAP-SREBP2 complex from the ER to the Golgi translocation process. Therefore, SCAP-SREBP2 plays an important role as a signaling hub for the integrated metabolism of cholesterol by macrophages and inflammation (176). In triple-negative breast cancer, the natural drug berberine inhibits the expression of NLRP3 (177).

In T-cells, FA metabolism is important in the development, differentiation, distribution, and function of different subsets of T cells (178). Cholesterol and phospholipids can be enriched around the immune synapse by lipid rafts to regulate the immune function of T cells (179). The aforementioned studies have shown the special role of lipids in immunity. SREBPs, as important regulators of lipid metabolism, act as a bridge between natural drugs and the treatment of tumors.

## Conclusion and Future Prospects

**FIGURE 4:**
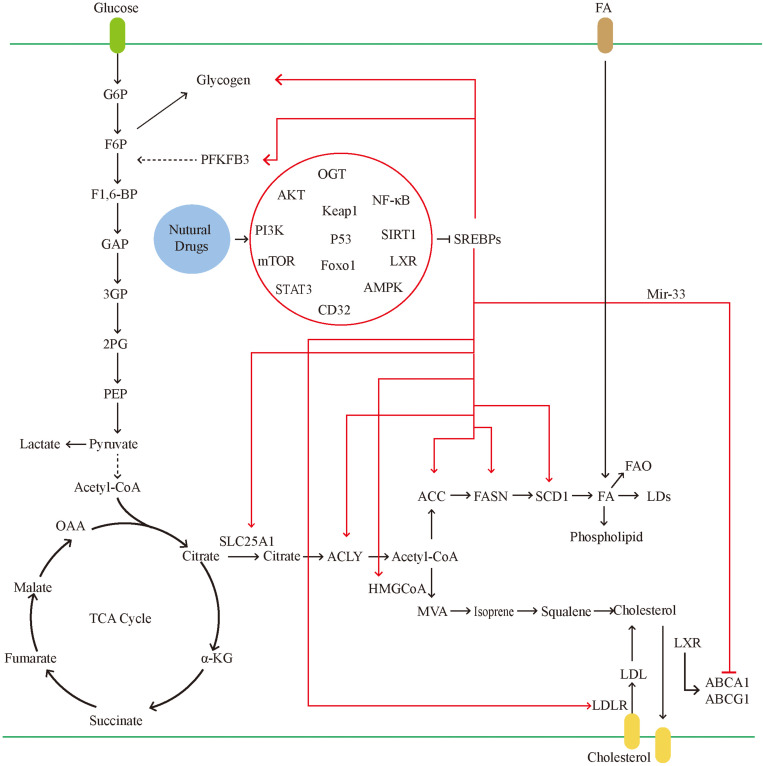
In tumor cells, the glucose uptake is significantly increased. SREBPs can promote glycogen production and accelerate glycolysis by activating the PFKFB. glycolysis leads to the massive production of pyruvate. Pyruvate can be converted into lactic acid and also participates in the tricarboxylic acid cycle. The tricarboxylic acid cycle produces citrate, which can be used as raw materials for lipid synthesis and cholesterol synthesis. Citrate transports mitochondria into the cytoplasm through SLC25A1. In tumors, activated SREBPs can promote the activity of multiple fatty acid synthases and accelerate the formation of fatty acids. Excess fatty acids can be converted into LDs and phospholipids. On the one hand, it is used for the proliferation of tumor cells and on the other hand to avoid lipotoxicity. SREBPs promote cholesterol absorption by activating LDLR, and mir-33, which is co-transcribed with SREBPs, can also inhibit the gene expression of transporters, thereby inhibiting cholesterol efflux. Natural drugs ultimately inhibit the activity of SREBPs through a variety of ways, including inhibiting gene transcription, reducing the transport of SREBPs, and reducing the maturation of SREBPs protein.

Sterol regulatory element-binding proteins, as key molecules for the traditional regulation of cellular lipid metabolism, have greatly expanded their range of capabilities in recent years (Figure 4). In the previous decades, research on SREBPs has gradually deepened. However, the mechanism through which SREBPs regulate lipid metabolism, affect other biological processes, and other factors targeting tumor cells remain to be fully understood. Although there are numerous drugs against SREBPs, their efficacy is limited and insufficient to transform the clinical treatment of tumors. This is attributed to the complex regulatory mechanisms of SREBPs. For example, SREBPs are well established as traditional lipid-regulating molecules, previous studies have suggested that almost all lipids are regulated by SREBPs. Nevertheless, current studies have found that the production of lipids also involves other pathways, such as protein kinase B. Induction of FA production is a mechanism independent of SREBP-1-mediated FA synthesis (180). This suggests that tumor cells can use a variety of ways to meet their energy requirements. At the same time, the regulation of target genes downstream of SREBPs can also be independent. For example, FASN can also regulate the production of lipids independently of SREBPs (54). The upstream mechanism regulating SREBPs is also diverse. Current research suggests that the feedback mechanism of ER is the main cause of dissociation of SREBPs and entry to the nucleus; however, the increase in SREBPs can also be cholesterol-insensitive. This suggests that, although SREBPs are extremely important in the metabolism of lipids, they are not required in special cases.

Primarily, the growth of tumor cells requires energy, glucose, environment, and other factors. SREBPs can provide abundant energy supply, abundant material reserves, excellent growth environment, and special protection for tumor cells. Many of these effects are dependent on the lipid metabolism regulated by SREBPs. Such complicated biological effects are strictly controlled by numerous mechanisms in the body; however, in tumor cells, this control is uncoordinated. Many drugs have demonstrated the unique ability and advantages of SREBPs.

To achieve better clinical results, we must re-examine the functions of SREBPs, and study the upstream and downstream mechanisms involved in these processes. It is also necessary to identify the specific mechanisms through which tumor cells regulate the elevation of SREBPs and ways to suppress this elevation. Combining targeting SREBPs with chemotherapy and immunotherapy is also a direction worthy of further consideration (27). For this purpose, natural medicine has already taken the lead, especially in the area of regulating lipid and glucose metabolism disorders, which is the pivotal cause of tumorigenesis. The signal pathways connecting lipid and glucose metabolism with tumors are the key targets of natural drugs or traditional Chinese medicines. Compounds exerting effects on the regulation of lipid or glucose metabolism, as well as inhibiting tumor growth, are candidates for the development of innovative anti-tumor drugs.

## Author Contributions

TJ prepared and edited the manuscript. ZL was responsible for modifying the manuscript. GZ provided some critical useful suggestions. All authors contributed to the article and approved the submitted version.

## Conflict of Interest

The authors declare that the research was conducted in the absence of any commercial or financial relationships that could be construed as a potential conflict of interest.
